# Combining globally search for a regular expression and print matching lines with bibliographic monitoring of genomic database improves diagnosis

**DOI:** 10.3389/fgene.2023.1122985

**Published:** 2023-04-20

**Authors:** Frédéric Tran Mau-Them, Alexis Overs, Ange-Line Bruel, Romain Duquet, Mylene Thareau, Anne-Sophie Denommé-Pichon, Antonio Vitobello, Arthur Sorlin, Hana Safraou, Sophie Nambot, Julian Delanne, Sebastien Moutton, Caroline Racine, Camille Engel, Melchior De Giraud d’Agay, Daphne Lehalle, Alice Goldenberg, Marjolaine Willems, Christine Coubes, David Genevieve, Alain Verloes, Yline Capri, Laurence Perrin, Marie-Line Jacquemont, Laetitia Lambert, Elodie Lacaze, Julien Thevenon, Nadine Hana, Julien Van-Gils, Charlotte Dubucs, Varoona Bizaoui, Marion Gerard-Blanluet, James Lespinasse, Sandra Mercier, Anne-Marie Guerrot, Isabelle Maystadt, Emilie Tisserant, Laurence Faivre, Christophe Philippe, Yannis Duffourd, Christel Thauvin-Robinet

**Affiliations:** ^1^ Unité Fonctionnelle Innovation en Diagnostic Génomique des maladies rares, CHU Dijon, Dijon, France; ^2^ INSERM UMR1231 GAD, Dijon, France; ^3^ Centre de Référence Maladies Rares “Anomalies du développement et syndromes malformatifs”, Centre de Génétique, FHUTRANSLAD et Institut GIMI, CHU Dijon Bourgogne, Dijon, France; ^4^ Normandie Univ, UNIROUEN, Inserm U1245 and Rouen University Hospital, Rouen, France; ^5^ Department of Genetics and Reference Center for Developmental Disorders, Normandy Center for Genomic and Personalized Medicine, Rouen, France; ^6^ Département de Génétique Médicale Maladies Rares et Médecine Personnalisée, Centre de Référence Maladies Rares Anomalies du Développement, Hôpital Arnaud de Villeneuve, Université Montpellier, Montpellier, France; ^7^ Centre de Référence Anomalies du Développement et Syndromes Malformatifs, Department of Medical Genetics, AP-HPNord- Université de Paris, Hôpital Robert Debré, Paris, France; ^8^ INSERM UMR 1141, Paris, France; ^9^ Service de Génétique Clinique, CHU Robert Debré, Paris, France; ^10^ Unité de Génétique Médicale, Pole Femme-Mère-Enfant, Groupe Hospitalier Sud Réunion, CHU de La Réunion, La Réunion, France; ^11^ Service de Génétique Clinique, CHRU Nancy, Paris, France; ^12^ Unité de Génétique Médicale, Groupe Hospitalier du Havre, Le Havre, France; ^13^ Département de Génétique, Assistance Publique-Hôpitaux de Paris, Hôpital Bichat, Paris, France; ^14^ INSERM U1148, Laboratory for Vascular Translational Science, Université Paris de Paris, Hôpital Bichat, Paris, France; ^15^ Service de Génétique Médicale, CHU de Bordeaux, Bordeaux, France; ^16^ Department of Medical Genetics, Toulouse University Hospital, Toulouse, France; ^17^ Service de Génétique, Centre Hospitalier Universitaire Caen Normandie, Caen, France; ^18^ Service de Génétique clinique, CH de Chambéry, Chambéry, France; ^19^ Service de Génétique Médicale, CHU Nantes, Nantes, France; ^20^ Department of Genetics and Reference Center for Developmental Disorders, Normandie Univ, UNIROUEN, CHU Rouen, Rouen, France; ^21^ Inserm U1245, FHU G4 Génomique, Rouen, France; ^22^ Centre de Génétique Humaine, Institut de Pathologie et de Génétique, Gosselies, Belgium

**Keywords:** GREP, intellectual disability, developmental anomalies, genomic database, diagnostic improvement, exome sequencing (ES), data reanalysis

## Abstract

**Introduction:** Exome sequencing has a diagnostic yield ranging from 25% to 70% in rare diseases and regularly implicates genes in novel disorders. Retrospective data reanalysis has demonstrated strong efficacy in improving diagnosis, but poses organizational difficulties for clinical laboratories.

**Patients and methods:** We applied a reanalysis strategy based on intensive prospective bibliographic monitoring along with direct application of the GREP command-line tool (to “globally search for a regular expression and print matching lines”) in a large ES database. For 18 months, we submitted the same five keywords of interest [(*intellectual disability*, (*neuro*)*developmental delay*, and (*neuro*)*developmental disorder*)] to PubMed on a daily basis to identify recently published novel disease–gene associations or new phenotypes in genes already implicated in human pathology. We used the Linux GREP tool and an in-house script to collect all variants of these genes from our 5,459 exome database.

**Results:** After GREP queries and variant filtration, we identified 128 genes of interest and collected 56 candidate variants from 53 individuals. We confirmed causal diagnosis for 19/128 genes (15%) in 21 individuals and identified variants of unknown significance for 19/128 genes (15%) in 23 individuals. Altogether, GREP queries for only 128 genes over a period of 18 months permitted a causal diagnosis to be established in 21/2875 undiagnosed affected probands (0.7%).

**Conclusion:** The GREP query strategy is efficient and less tedious than complete periodic reanalysis. It is an interesting reanalysis strategy to improve diagnosis.

## Introduction

Exome sequencing (ES) is now used for routine diagnostic testing. ES has a diagnostic yield ranging from 25% to 70% (Stark et al., 2016; Tan et al., 2017; Clark et al., 2018), depending on the type of disorder, the presence of consanguinity, and the strategy used (solo or trio). Clinical laboratories have rapidly developed an attraction to the substantial benefits of data reanalysis, since ES regularly identifies causative variants in a large number of genes responsible for ultra-rare Mendelian disorders not yet associated to human disorders, particularly in highly heterogeneous diseases such as developmental disorders (DDs) or intellectual disability (ID) (Boycott et al., 2017; Wenger et al., 2017; Hartley et al., 2020).

The strategy of ES data reanalysis for undiagnosed individuals has demonstrated high efficiency. Complete retrospective reanalysis of clinical ES data after a defined period of time has elapsed, along with pipeline updates (especially to the OMIM and ClinVar databases), leads to an additional diagnostic yield ranging from 10.5% to 32% (Table 1) (Costain et al., 2018; Ewans et al., 2018; Nambot et al., 2018; Baker et al., 2019; Li et al., 2019; Salfati et al., 2019). A recent review of published reanalysis papers reported 10% diagnostic yield, but with considerable heterogeneity due to the delay in reanalysis (after or before 24 months), which is the limitation to OMIM-morbid genes or research extension (Dai et al., 2022). The major reasons for novel diagnosis are not only the ongoing discovery of novel genes involved in human diseases (Boycott et al., 2017) but also novel annotations of well-known OMIM-morbid genes extending their clinical phenotypes (Fokstuen et al., 2016). However, genes that are newly associated with human disorders are not instantly implemented in the commonly used public databases (OMIM, ClinVar, etc.) or in laboratory databases, which limits the power of reanalysis in the diagnostic setting. Diagnostic yield may indeed be found to increase significantly, from 30% to more than 40% in DD/ID, when reanalysis is extended to translational research involving data-sharing for candidate genes (Bruel et al., 2019). Because systematically performing complete reanalysis of ES data represents a significant challenge for clinical laboratories, a semi-automated reanalysis pipeline that interrogates various databases could facilitate efficient re-evaluation of undiagnosed individuals using up-to-date literature; this could be of significant value to clinical laboratories (Costain et al., 2018).

**TABLE 1:** Manuscripts focusing on a reanalysis strategy with diagnostic yield and delay. ES: exome sequencing; GS: genome sequencing; NR: not reported.

Publication	Total diagnostic yield after reanalysis in % (N)	Delay after first-tier test (type of test)	Diagnostic rate in %	Diagnostic rate per year in %
Nambot et al. (2018)-7	15.4 (24/156)	24 months (solo ES)	15.4	7.7
Wenger et al. (2017)-6	10 (4/40)	20 months on average (NR)	NR	NR
Costain et al. (2018)-11	10.9 (7/64)	2 years on average (solo GS)	NR	NR
Ewans et al. (2018)-9	10.8 (6/54)	12 months (solo and trio ES)	10.8	10.8
Li et al. (2019)-8	10.5 (8/76)	0–6 months; 6 months–1 year; >1 year (trio ES)	15; 14.3; 3.6	NR
Baker et al. (2019)-10	15.8 (38/240)	>10 months (solo, duo, and trio ES)	NR	6.84

In addition to reanalysis of complete ES data, several targeted strategies can be applied on request. ES data can thus be interrogated using GREP, a command-line tool used to “globally search for a regular expression and print matching lines” that makes it possible to search for specific expressions in files (https://www.gnu.org/software/grep/manual/grep.html). By default, a GREP query will search for lines containing a given string pattern in a file or the standard input, but the query can be customized for enhanced specificity. The GREP command-line tool has been used in cancer applications to look for gene fusion or Alu insertion in unique patients or cohorts with similar disorders (Panagopoulos et al., 2014; Bujakowska et al., 2015; Panagopoulos et al., 2015). After identifying a balanced translocation t(10; 17) (q22; q21) following the use of cytogenetic techniques in an affected individual with retroperitoneal leiomyoma, Panagopoulos et al. (2015) identified a breakpoint in exon 3 of *KAT6B*, localized in the 10q22.2 region. A GREP query of this *KAT6B* exon sequence identified a unique chimeric sequence of 101 nucleotides composed of 43 nucleotides from this exon and 60 nucleotides from the *KANSL1* gene, localized in the 17q21 region. A similar approach was applied to a large cohort following the identification by cytogenetic techniques of a balanced translocation t(4; 19) (q35; q13) in an affected individual with small round cell sarcoma. The same group identified a *CIC–DUX4* fusion transcript that was not detected by several other algorithms designed to identify fusion (Panagopoulos et al., 2014). A GREP query on a known junction sequence of an Alu insertion in *MAK* was applied in a cohort of 1,847 samples (data from targeted sequencing or ES) of individuals with retinitis pigmentosa; this enabled the discovery of five affected individuals with the same GREP term (Bujakowska et al., 2015). In rare diseases, targeted GREP queries can be performed over ES or GS data on request and/or for specific purposes (Panagopoulos et al., 2014; Bujakowska et al., 2015; Panagopoulos et al., 2015). Nevertheless, intensive use of GREP query strategies for certain genes or variants has never been reported.

We present an innovative reanalysis strategy combining intensive prospective medical bibliographic monitoring with subsequent use of rapid GREP querying in a large ES database and international data-sharing to improve diagnostic yield and reduce diagnostic delay in individuals with DD/ID.

## Patients and methods

### Patients

Since 2013, our clinical laboratory has performed ES in 5,459 individuals (4,170 probands and 1,289 affected or unaffected relatives), referred by several French university hospitals. Among the 4,170 probands, 3,771 were referred for DD and/or ID; 896/3,771 (23.7%) probands in this DD/ID group had a positive molecular diagnosis and 2875/3,771 (76.3%) had no causal diagnosis. Among the relatives, 98 belonged to a cutaneous mosaicism group, 23 to a cancer group, and 1,168 to the diverse DD/ID group. The ES methods employed have been previously reported (Tran Mau-Them et al., 2021).

All patients were informed of the continuous updating of their genomic data with regard to advances in knowledge and of the fact that they could be contacted again in the future in the event of the identification of new results useful for their health. All patients gave their informed consent for this procedure.

### Prospective medical bibliographic monitoring

From April 2019 to October 2020, manual monitoring of medical bibliographies was performed every day by one medical biologist. The biologist searched the PubMed database for the following terms: *intellectual disability*, (*neuro*)*developmental delay*, and (*neuro*)*developmental disorder*. The PubMed search output was sorted using the “most recent” display option, and only the 10 articles displayed on the first page were considered. When an article described a novel gene–disease association or broadened a known gene–disease phenotype, we considered this article to be relevant. In addition, the gene name was searched on OMIM to look for any existing association with human disorders. Finally, the gene in question was searched in the ES database using the GREP command-line tool.

### GREP strategy

Use of the daily GREP query strategy started in April 2019, with a basic GREP query used to search for every rare variant (variant allele frequency <1%) of genes of interest in all the vcf files available (hg19). These vcf files are text files storing gene sequence variants with a mandatory minimum of eight columns and custom annotation. The custom annotation included general population frequency and occurrence [in the Exome Sequencing Project (ESP), Exome Aggregation Consortium (ExAC), Genome Aggregation Database (gnomAD) exome/genome, and a control sample count from our own database]; a batch sample count; several pathogenicity scores [Polyphen (Adzhubei et al., 2010), GERP (Cooper et al., 2005), Grantham (Grantham, 1974), CADD (Kircher et al., 2014), misZ, and pLI]; the OMIM pathology name, number, and mode of transmission; and lastly, the ClinVar classification. This basic GREP query performed by the biologist is referred to as v1 in the manuscript figures and tables. For example, the basic GREP query “grep -w GENE1 *.tsv > GENE1.tsv” searches for the exact expression “GENE1” in all files ending with “.tsv” and creates a new file “GENE1.tsv”. The -w option is included so that the query searches for whole-word matches for GENE1 and will not print lines such as GENE10, GENE11, etc.

**FIGURE 1:**
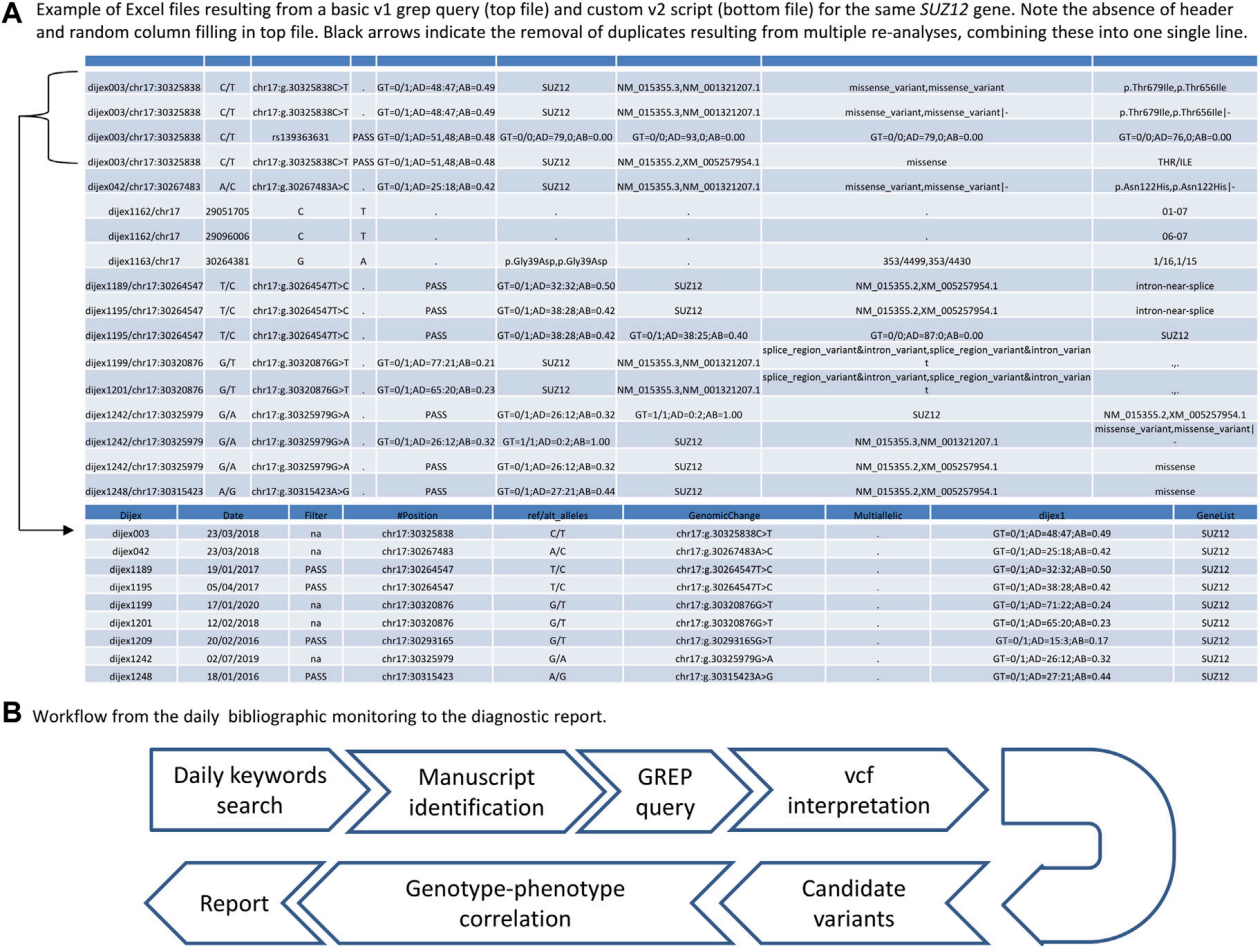
**(A)** Tabular files produced by a basic GREP query (top) and custom script query (bottom) for *SUZ12*. Note the absence of headers, the duplicate lines, and the disordered columns in the file resulting from the basic GREP query, which are corrected in the version output by the in-house script. **(B)** Workflow of the fast GREP query strategy.

With the increasing amount of ES data (novel or reanalysis) available, the number of lines printed increases accordingly, leading to hardly readable files. To improve this command-line tool and make the resulting file more biologist-friendly, in early 2020, we developed a bash script set up for our specific working environment, referred to as v2. The novel command-line script only requires the gene name as an argument, meaning that the biologist only has to type “GENE1” (example query: “getthatgene.bash GENE1”, compared to the more complex v1 GREP query*.* The output of the v2 GREP query was reformatted using a python3 script called by the bash script. The most important steps included 1) removal of exome reanalysis duplicates to retain only the most recently analyzed file, and 2) filling of gaps in the additional annotations with dots to produce an intelligible spreadsheet (Figure 1A). Three novel annotations were added during versioning of the database, namely, the and observed/expected with lower/upper threshold, Splicing Prediction Pipeline, (Leman et al., 2022) and the CCR (Havrilla et al., 2019) scores. Finally, in October 2020, we performed an end-point GREP query by searching again for all the genes previously searched with the daily GREP queries to see whether we could recover any missed diagnoses due to the time that had elapsed between the initial daily GREP queries and the end-point GREP query.

A positive GREP query was defined by the identification of a candidate variant and a negative GREP query by its absence.

Although our ES analysis currently includes SNVs, CNVs, and mitochondrial variants from off-target sequences (Garret et al., 2019), only rare SNVs from nuclear genomes were searched for using these command line queries.

### Variant interpretation and classification

For all variants recovered using the GREP queries, interpretation was focused on the same variant type (missense variants in the same protein domain or protein-truncating variants) and mode of inheritance as reported in the manuscripts from PubMed. For the remaining candidate variants of interest, patient phenotypes were compared to the new disease–gene associations; only variants identified in the 3,771 individuals with DD/ID and their 1,168 relatives were considered. Candidate variants (*i.e.*, those with compatible genotype–phenotype correlation) were confirmed using a second independent method (Sanger sequencing or quantitative PCR) and then shared via international collaborative platforms (GeneMatcher) (Sobreira et al., 2015) to strengthen the community’s knowledge of genotype–phenotype correlations since only one article had reported these new disease–gene associations in most cases. The workflow for the rapid GREP query strategy is summarized in Figure 1B.

Variant classification was based on the ACMG–AMP classification (Richards et al., 2015).

## Results

### Prospective medical bibliographic monitoring

**FIGURE 2:**
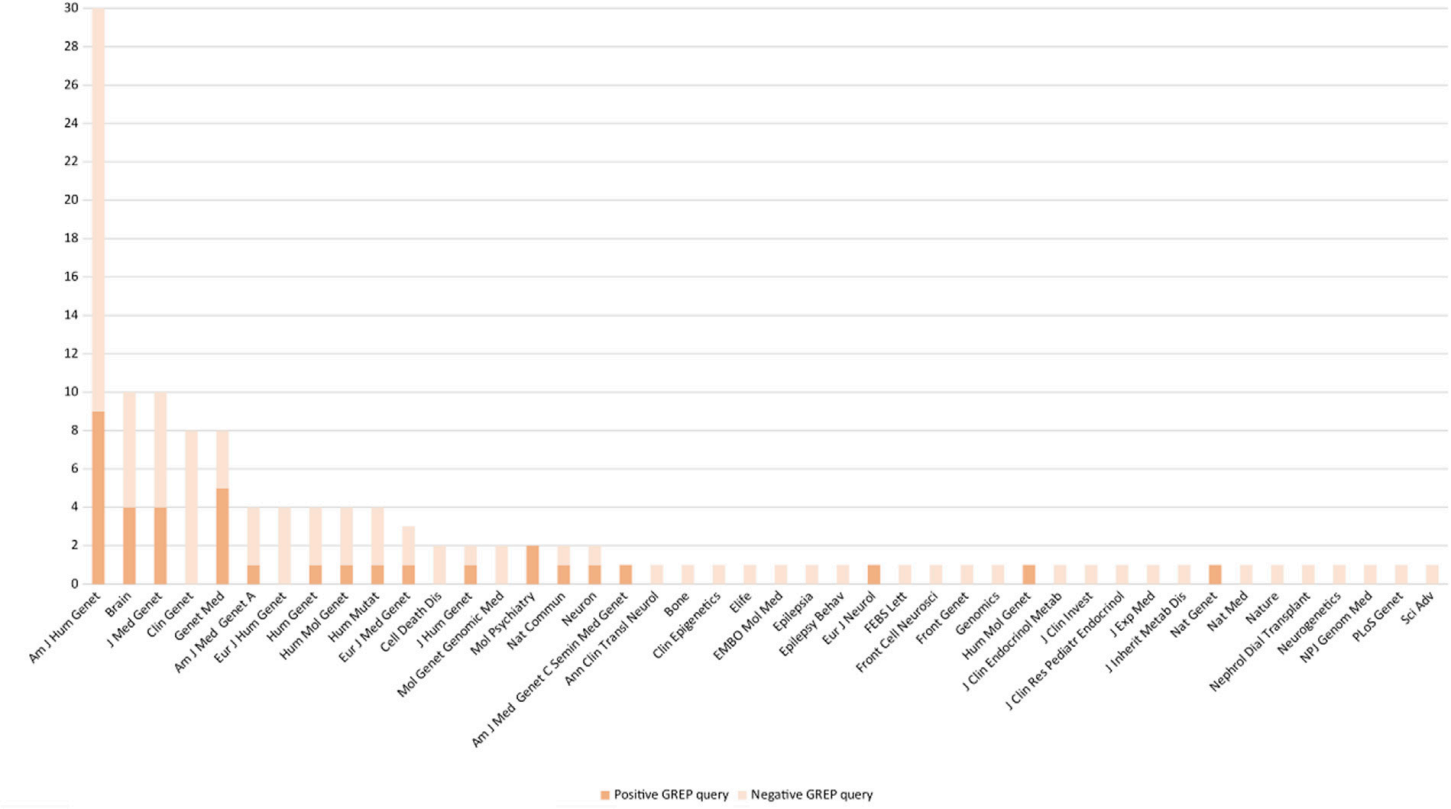
Number of positive and negative GREP queries arising from manuscripts published in various journals.

**FIGURE 3:**
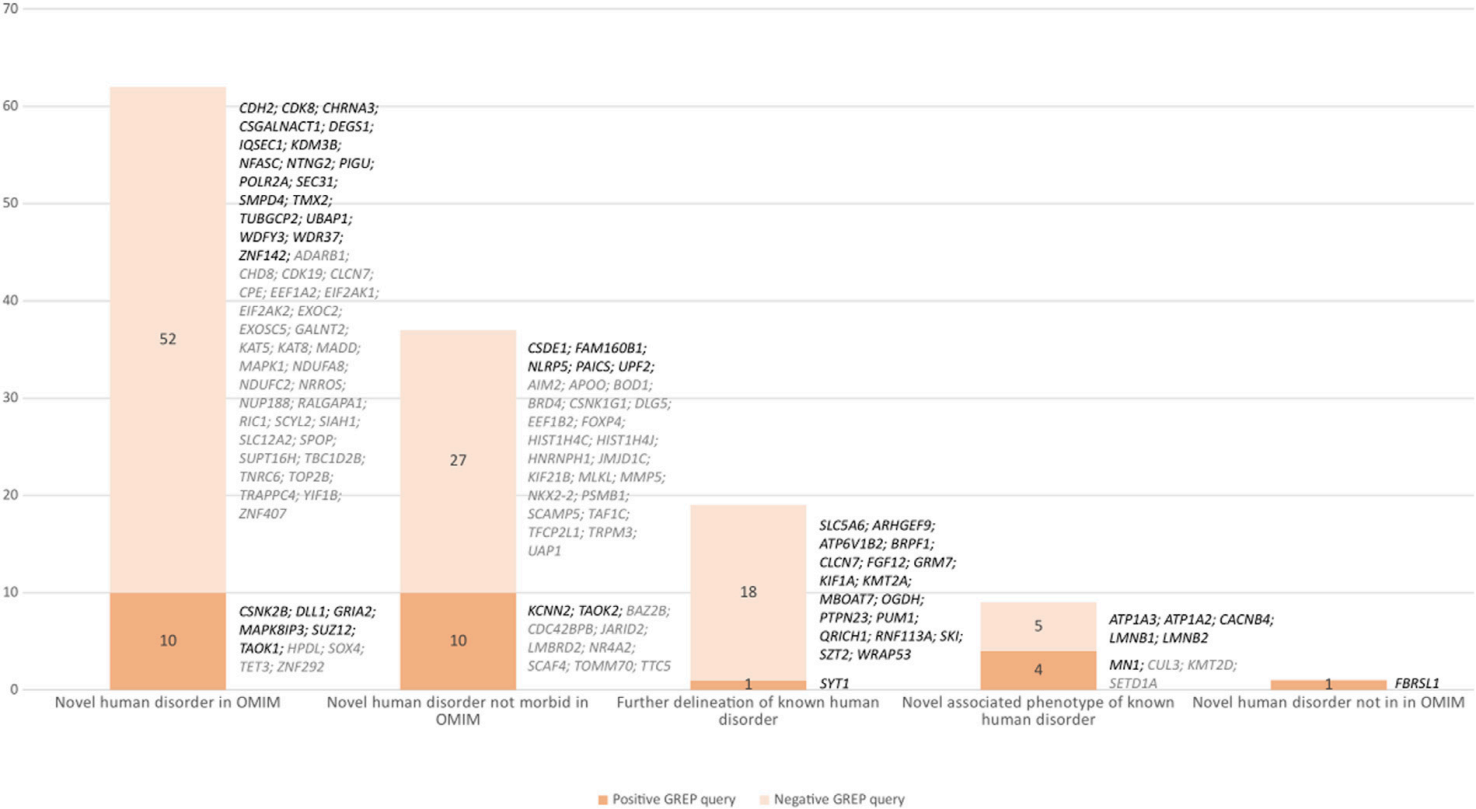
Number of genes resulting from positive and negative GREP queries with different levels of association with human disorders. Black indicates a GREP query run in 2019 and gray a query run in 2020. Evolution of the number of exomes in the database and number of genes queried with positive and variants of unknown significance patients.

**FIGURE 4:**
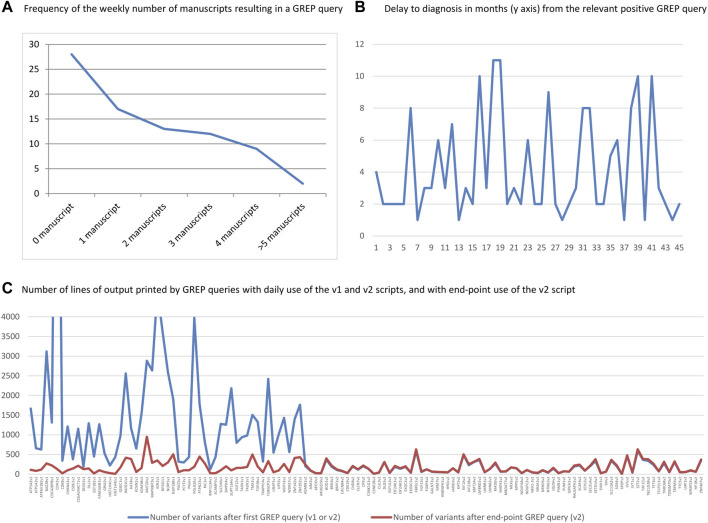
**(A)** Number of weeks with the specified number of manuscripts resulting in a GREP query. **(B)** Delay in months between positive GREP query and diagnostic report. **(C)** Number of lines of output printed by GREP queries for each of the 128 genes, for daily queries using the v1 and v2 scripts and end-point GREP queries using the v2 script. Note the drastic difference between v1 and v2.

From April 2019 to October 2020, prospective medical bibliographic monitoring identified 128 genes implicated in DD/ID, mainly from publications in the *American Journal of Human Genetics* (30/128 genes), the *Journal of Medical Genetics* (10/128 genes), *Brain* (10/128 genes), *Clinical Genetics* (8/128 genes), and *Genetics in Medicine* (8/128 genes) (Figure 2; Supplementary Table S1). Among these 128 genes, 100 were associated with a novel human disorder (of which 37 were still not classified as morbid in the OMIM database and one was not reported in OMIM at all), nine had already been associated with a distinct human disorder, and 19 represented updates to the genotype/phenotype correlation of a known human disorder (Figure 3). The number of relevant manuscripts identified per week ranged from 0 to more than 5 (Figure 4A).

### GREP strategy

The time interval between a positive GREP query and delivery of the diagnostic report to the clinician ranged from 1 to 11 weeks (overall mean and SD: 4.2 ± 3.1; mean in 2019: 3.4 ± 2.2; mean in 2020: 4.5 ± 3.5; Figure 4B). For the daily GREP queries, the average time was 2.6 months (SD ± 1.8), compared with 6.6 months (SD ± 3.4) for the end-point GREP queries.

We also calculated the number of printed lines resulting from GREP queries using v1 and v2. Overall, the v1 script resulted in 52 genes and 82,156 lines of output (mean: 1,579 output lines/gene) and the v2 script resulted in in 76 genes and 11,953 lines of output (mean: 157 output lines/gene). This represented a drastic reduction (mean: 10-fold) in the number of output lines between v1 and v2. There was a slight increase in the number of lines of output between the two sets of v2 searches (mean difference: 1.1 times) due to the increase in the number of exomes in the database between the first v2 queries and the end-point GREP queries (Figure 4C). This use of the GREP query retrieved 36 genes in 2019 (starting from April) and 92 genes in 2020 (from January to October), for ES data covering a grand total of 5,459 individuals.

### Molecular results

**FIGURE 5:**
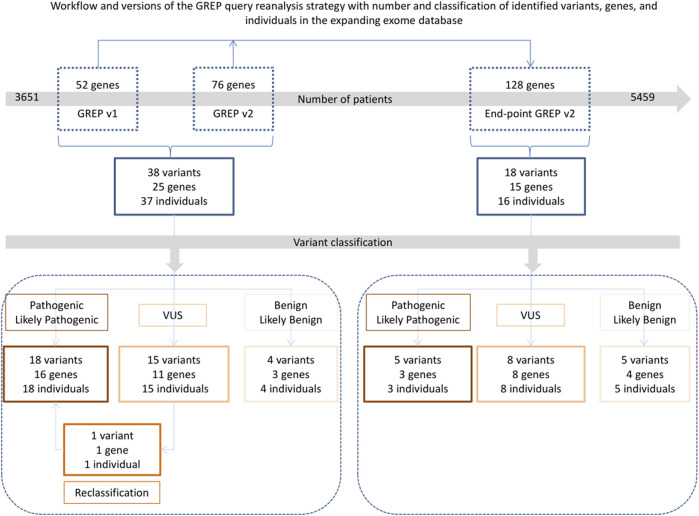
Workflow and version of GREP query used according to the amount of ES data searched, with the number and classification of identified variants, genes, and individuals. A *CSNK2B* variant was first classified as of unknown significance but subsequently reclassified as likely pathogenic after a later publication. VUS: variant of unknown significance.

From the daily GREP queries, we obtained results of interest for 38 variants (29 premature stop codons and nine missenses) in 25/128 genes (20%) in 37 individuals (Figure 5). After Sanger validation and family segregation, 18 variants were classified as pathogenic or likely pathogenic (P/LP), 15 as variants of unknown significance (VUS), and four as benign or likely benign (B/LB). In three genes, we identified both P/LP variants and VUS (*MAPK8IP3*, *SCAF4*, and *SUZ12*), and in one gene, we identified P/LP, VUS, and B/LB variants (*ZNF292*) (Table 2).

**TABLE 2:** Candidate variants identified using GREP queries, with classification after parental segregation. NA: not available; P/LP: pathogenic/likely pathogenic; VUS: variants of unknown significance; LB: likely benign.

Gene	Delay to diagnostic report (months)	Variant segregation	Genomic position	Protein	Number of patients	P	LP	VUS	LB	ACMG–AMP retrospective criteria
*CSNK2B*	4	*De novo*	chr6:g.31637615T>C	p.(Leu187Pro)	1		+			VUS (PM1, PM2, PP5, and BP1)
*DLL1*	2	*De novo*	chr6:g.170594361del	p.(Lys338Argfs*28)	1		+			NA
*GRIA2*	2	*De novo*	chr4:g.158282245G>A	p.(Gly792Glu)	1		+			LP (PM1, PM2, PM5, and PP2)
*KCNN2*	2	*De novo*	chr5:g.113808863T>C	p.?	1			+		NA
*MAPK8IP3*	2	*De novo*	chr16:g.1812844C>T	p.(Arg578Cys)	2	+				LP (PM1, PM2, PP3, and PP5)
	8	ND	chr16:g.1813786T>G	p.?				+		NA
*MN1*	1	*De novo*	chr22:g.28147072del	p.(Pro1265Leufs*55)	1	+				NA
*NFASC*	3	Paternally and maternally inherited	chr1:g.204943318C>T	p.(Arg431Trp)	1			+		VUS (PM1)
*SMPD4*	3	Maternally inherited	chr2:g.130914172del	p.Ala431Hisfs*9	1		+			NA
		Paternally inherited	chr2:g.130914204_130914206del	p.Phe419del			+			NA
*SUZ12*	6	Maternally inherited	chr17:g.30320326C>T	p.(Arg423*)	2		+			NA
		Paternally inherited	chr17:g.30264544G>A	p.?				+		NA
*TAOK1*	3	Maternally inherited	chr17:g.27816684G>T	p.(Glu220*)	1		+			NA
	7	*De novo*	chr17:g.27809240A>C	p.(Met197Leu)	1		+			VUS (PM1 and PM2)
*TAOK2*	1	Paternally inherited	chr16:g.29998795A>T	p.(Arg1068*)	1			+		NA
*WDFY3*	3	Paternally inherited	chr4:g.85687036_85687037del	p.(Lys1705Argfs*10)	1		+			NA
*BAZ2B*	2	Maternally inherited	chr2:g.160287462dup	p.(Ser703Leufs*9)	1			+		NA
BRD4	10	*De novo*	chr19:g.15374283T>C	p.(Tyr430Cys)	1		+			VUS (PM1, PM2, and PP2)
*CDC42BPB*	3	Maternally inherited	chr14:g.103440469G>A	p.(Arg509*)	3			+		NA
	11	Paternally inherited	chr14:g.103410506T>C	p.Gln1377Arg					+	VUS (PM1 and PM2)
	11	Paternally inherited	chr14:g.103432620A>T	p.Leu859Gln					+	VUS (PM2)
*CUL3*	2	*De novo*	chr2:g.225368388dup	p.(Asn453Lysfs*5)	3			+		NA
		Maternally inherited	chr2:g.225365152C>T	p.(Trp513*)				+		NA
	3	Maternally inherited	chr2:g.225370672C>A	p.?				+		NA
*FBRSL1*	2	Paternally inherited	chr12:g.133158105G>T	p.?	2				+	NA
	6	ND	chr12:g.133067271G>T	p.(Glu39*)					+	NA
*HPDL*	2	Paternally and maternally inherited	chr1:g.45793608C>T	p.(Thr263Met)	2		+			VUS (PM1, PP3, and PP5)
			chr1:g.45793162_45793165dup	p.(Ala116Cysfs*81)			+			NA
*JARID2*	2	Maternally inherited	chr6:g.15501555G>A	p.(Arg788Gln)	1			+		VUS (PM1, PP3, and BP1)
*JMJD1C*	9	Maternally inherited	chr10:g.64974008del	p.(Pro640Hisfs*10)	1			+		NA
*KMT2D*	2	Maternally inherited	chr12:g.49427884T>C	p.(Glu3569Gly)	1		+			VUS (PM2 and BP1)
*LMBRD2*	1	*De novo*	chr5:g.36115212G>A	p.(Arg483Cys)	1		+			VUS (PM2 and PP5)
LMNB1	2	Maternally inherited	chr5:g.126140563C>T	p.(Ala152Val)	1				+	LP (PM1, PM2, and PM5)
MPP5	3	Maternally inherited	chr14:g.67779336A>G	p.(Ile378Met)	1				+	VUS (PM1, PM2, and BP4)
*NR4A2*	ND	Not maternally inherited	chr2:g.157186374del	p.(Gln109Serfs*5)	1		+			NA
*NUP188*	8	Paternally and maternally inherited	chr9:g.131745626del	p.(Cys617Trpfs*2)	1		+			NA
			chr9:g.131760903G>A	p.?			+			NA
*SCAF4*	2	Paternally inherited	chr21:g.33065654_33065657del	p.(Arg488Asnfs*10)	2			+		NA
		Maternally inherited	chr21:g.33043941_33043944del	p.(Glu1071Glyfs*12)				+		NA
*SETD1A*	2	ND	chr16:g.30976565del	p.(Lys502Serfs*159)	1			+		NA
*SLC12A2*	5	Paternally inherited	chr5:g.127512826C>G	p.(Gln987Glu)	1				+	VUS (PM1, PM2, and BP1)
*SOX4*	6	Paternally inherited	chr6:g.21595127C>G	p.Ala121Gly	1			+		LP (PM1, PM2, PP2, and PP3)
*SYT1*	1	Maternally inherited	chr12:g.79837973T>A	p.(Phe350Tyr)	1			+		VUS (PM1, PM2, and PP2)
*TET3*	ND	Paternally inherited	chr2:g.74314995C>A	p.(Cys906*)	1		+			NA
	8	Paternally inherited	chr2:g.74320729C>G	p.(Ala1068Gly)	1				+	VUS (PM1 and PM2)
*TNRC6B*	10	Paternally inherited	chr22:g.40696947C>T	p.Gln1292*	1			+		NA
*TOMM70*	1	Paternally inherited	chr3:g.100086949T>C	p.(Ile538Val)	1				+	VUS (PM1 and PM2)
	10	Maternally inherited	chr3:g.100087956del	p.(Phe492Leufs*112)	1			+		NA
*TTC5*	3	Paternally inherited	chr14:g.20757884G>A	p.(Arg409*)	1		+			NA
		Maternally inherited	chr14:g.20763470C>T	p.?			+			NA
*ZNF292*	2	*De novo*	chr6:g.87969507_87969508del	p.(Glu2054Lysfs*14)	6		+			NA
		*De novo*	chr6:g.87966666C>T	p.(Arg1107*)			+			NA
	1	Maternally inherited	chr6:g.87968280C>T	p.(Gln1645*)				+		NA
	2	Probable paternal inheritance	chr6:g.87925776G>A	p.?				+		NA
		Not maternally inherited	chr6:g.87970807del	p.(Leu2487Cysfs*6)				+		NA
		Maternally inherited	chr6:g.87970961del	p.(Asn2538Lysfs*21)					+	NA

All 18 P/LP variants in the 16 genes were identified in 18 previously undiagnosed DD/ID affected individuals, with diagnostic odysseys ranging from 6 months to 6 years. Thirteen of the 16 genes had not previously been reported to be involved in human disorders in the OMIM database and therefore were not annotated in the pipeline and had not been considered in the first diagnostic analysis. The other three genes were known to be involved in human disorders with an OMIM number, but with a different phenotype (*KMT2D*, *MN1*, and *SETD1A*). In one of the individuals, a patient with epileptic encephalopathy and a family history of long QT and with a previously identified *KCNQ1* pathogenic variant, we also identified an *HPDL* causative variant [p.(Ala116Cysfs*81)], leading to a dual diagnosis. The GREP queries enabled the reclassification as likely pathogenic of two variants initially classified as VUS by the solo ES analysis (*NR4A2* and *TET3*).

Fifteen variants (12 truncating and three missense) were classified as VUS, mainly because they were inherited from asymptomatic parents (10/15 individuals), a situation not described in the original manuscripts (Table 2). For 3/15 VUS cases, the parental segregation was not available (*MAPK8IP3* and *ZNF292*). One of the 15 VUSs was secondarily reclassified as likely pathogenic after the publication of additional data 7 months after the initial GREP query (*CSNK2B*). One of the 15 VUSs was a *de novo* truncating variant in *CUL3*. Two VUSs were identified in individuals with previously identified causative variants: an inherited *SCAF4* truncating variant [p.(Glu1071Glyfs*12)] in a fetus carrying a pathogenic *IGF2* variant, and an inherited *SYT1* missense variant [p.(Phe350Tyr)] in an individual carrying a *SRCAP* pathogenic variant. For 6/11 genes, international collaborations are ongoing to better characterize the genotype/phenotype of the affected individuals and could lead to reclassification of these variants (*BAZ2B*, *CUL3*, *SCAF4*, *SUZ12*, *TAOK2*, and *ZNF292*) as causative.

After the end-point GREP queries, we identified additional results of interest for 18 variants (10 missense, seven truncating, and one in-frame deletion) in 11 additional genes and four variants in genes with previous positive GREP query results, in 16 additional individuals (Figure 5; Table 2). After Sanger validation and family segregation, five variants were classified as P/LP (in three genes for three affected individuals), eight as VUS (in eight genes for eight affected individuals), and five as B/LB (in four genes for five affected individuals). The five P/LP variants identified the three genes had not been considered in the initial analysis for two reasons. First, the queries for *SMPD4* and *WDFY3* were carried out using the v1 script, probably leading to output that was too complicated for interpretation (1,259 variants in the v1 GREP query for *SMPD4* and 1,433 for *WDFY3*). Second, the query for *NUP188* was carried out in February 2020 using the v1 script, but the individual in question was only added to our exome database in August 2020, so the variants could not have been detected previously. They would also not have been detected in a diagnostic setting, since this gene was not implemented as an OMIM-morbid gene at this time.

After the end-point GREP queries, additional genes were found to harbor additional candidate variants, namely, *TAOK1* (P/LP variants and VUS), *CDC42BPB* and *TOMM70* (VUS and B/LB variants), and *TET3* (P/LP and B/LB variants).

Among the five P/LP variants identified in the end-point GREP queries, four were identified in two previously undiagnosed individuals with DD/ID, with diagnostic odysseys ranging from 4 months (*NUP188*) to 3 years (*SMPD4*). In one of the three individuals with an overgrowth history and a previously identified pathogenic CNV in chromosome 16 (containing *TAOK2*), we also identified a *WDFY3* causative variant [p.(Lys1705fs)], leading to a dual diagnosis. Eight variants (five missense and three truncating) identified in the end-point GREP queries were classified as VUS because they were inherited from asymptomatic parents in 6/8 individuals, which was not as described in the original manuscript. For two *de novo* variants, international collaborations are ongoing to better characterize the genotype/phenotype of the affected individuals and could lead to reclassification of these variants (*BRD4* and *TAOK1*) as causative.

Altogether, we identified 56 variants in 36 different genes in 53 affected individuals by performing the daily and end-point GREP queries (Figure 5; Table 2). Moreover, all the VUSs could be considered likely pathogenic because they harbored the same type (protein truncating variant or missense located in the same domains) as in the relevant published manuscript.

## Discussion

This study is the first to present the feasibility and value of a reanalysis strategy combining intensive medical bibliographic monitoring with the use of a rapid GREP query applied to large ES data for the diagnosis of individuals with DD/ID. Altogether, this strategy identified 56 variants in 36 different genes in 53/3,771 affected probands (1.4%), including a causative variant in 21/53 (39.6%), rising to 44/53 (83%) when considering reclassified variants and VUS. Therefore, GREP queries for only 128 genes during a period of 18 months permitted a causal diagnosis to be established in 21/2,875 undiagnosed affected probands (0.7%). This yield is probably an underestimate because the initial cohort was heterogeneous and because the use of only a few keywords [ID, (neuro)developmental delay/disorder] will not have encompassed the full spectrum of the cohort (10% being unaffected by ID/DD). Moreover, with the majority of the candidate genes being responsible for ultra-rare diseases, this strategy would probably prove to be more effective if the number of undiagnosed affected individuals was much greater.

The novel diagnoses were mainly established in genes newly implicated in human diseases (32/36 genes). These genes had not been reported to be involved in human disorders in the OMIM database at the time of the initial clinical ES analysis and thus, could not be retained by the usual diagnostic analysis. Since these variants were present in the vcf files used in the initial ES analysis, a translational research analysis extended to non-OMIM (morbid) genes could focus on these candidate genes (Fokstuen et al., 2016). However, some variants appear to be very difficult to interpret, especially missense variants [39% of variants (22/56) identified in our GREP queries]. Moreover, all the VUSs could be considered likely pathogenic because they harbored the same type (protein truncating variant or missense located in the same domains) as in the relevant published manuscript. However, in most cases, with only one manuscript reporting these variants, additional observations of genotype–phenotype correlation are warranted to definitively implicate these genes and variants in human disorders. Most of these variants have been shared through various data-sharing systems, leading to ongoing international collaborations with the aim of further characterizing the genotype–phenotype correlation and/or conducting functional analysis. As expected, one category of variants remains easier to interpret in research analysis, *i.e*., truncating variants in genes with pLI > 0.9 or o/e < 0.3 (66% of variants identified in our GREP queries).

In a very small number of genes (4/36), variants were identified in well-known OMIM-morbid genes (*CUL3*, *KMT2D*, *MN1*, and *SETD1A*) but had not been initially considered in the first analysis because the phenotype of the referred individual and/or the mechanism of the variant was completely different from what was known. For instance, heterozygous *CUL3* variants have been associated with pseudohypoaldosteronism type IIIE (MIM # 614496) due to an in-frame deletion of exon 9, and only recently have loss-of-function (LoF) variants been found to be involved in overall developmental delay (Nakashima et al., 2020). Heterozygous LoF variants in *KMT2D* are involved in Kabuki syndrome type 1 (MIM #147920), but specific heterozygous variants in exons 38 and 39 are likely to act in a dominant negative mechanism (Cuvertino et al., 2020). Fusion transcripts in *MN1* are involved in meningioma (MIM # 607174), but specific truncating variants are thought to act in a dominant negative mechanism (Mak et al., 2020; Miyake et al., 2020). Missense variants outside protein domains in *SETD1A*, and of unclear effect, have been associated with early-onset epilepsy with or without ID (MIM #618832) (Yu et al., 2019), whereas LoF variants have been associated with a novel neurodevelopmental syndrome (MIM #611052) (Kummeling et al., 2021).

The major aspect of interest of this strategy remains the rapid translation from published results in PubMed to a diagnostic report, with a mean time between online publication in PubMed and diagnostic report of 4.2 months on average (SD ± 3.1). Indeed, the total number of genes found to be newly involved in human disorders represents a mean of 38 (31–49) genes per month (i.e., one gene per day) in 2019/2020 according to OMIM statistics (Boycott et al., 2017). However, the time that elapses between online publication in PubMed and OMIM indexing can be up to several months, which delays the annotation of these genes and hampers their interpretation in clinical routine practice. It also requires periodic pipeline updates with information from different databases (OMIM, ClinVar, HGMD, and denovoDB) and reanalysis to improve diagnosis. Despite their recognized utility (Nambot et al., 2018; Bruel et al., 2019), periodic reanalysis strategies in diagnostic and research settings may represent a significant challenge for clinical laboratories. These appear to be very time-consuming, with systematization being possible only if a sufficiently large translational research team is available to work in partnership with the clinical laboratory and physicians. A strategy combining intensive prospective bibliographic monitoring and targeted GREP queries appears to be a good compromise for the workload of clinical laboratories, especially since the usual diagnostic pipelines rely on updated data to aid in the interpretation of genes newly found to be involved in human disorders and/or flagged with pathogenic information in databases. In addition, this strategy enables faster diagnoses than periodic reanalysis. The time delay appears to be significantly reduced in the daily strategy, compared to published periodic reanalysis strategies, where it currently ranges from 6 to 18 months (Figure 1A) (Costain et al., 2018; Ewans et al., 2018; Nambot et al., 2018; Baker et al., 2019; Li et al., 2019; Salfati et al., 2019) or may be conducted on physicians’ request. Nevertheless, manuscript selection via intensive medical bibliographic monitoring could also be time-consuming for biologists. Ideally, this strategy should be combined with periodic reanalysis since we cannot guarantee that all novel implications and phenotypes have been investigated, despite thorough bibliographic monitoring.

To monitor relevant bibliographies, we periodically performed searches on PubMed using five relevant clinical keywords for DD/DI. Among the 128 genes retained in the results of the GREP queries, 66/128 (51.5%) were from articles published in five different journals in the field of human genetics (*Am J Hum Genet; Brain; J Med Genet; Clin Genet;* and *Genet Med*) (Supplementary Table S1). To improve the strategy, the choice of keywords for literature monitoring is essential. They must belong to the clinical area of the cohort studied. Indeed, our keywords were suitable for DD/DI but did not capture a wide range of genetic diseases and would not be suitable for other databases. Limiting their number or combining them would reduce the chances of identifying suitable candidate genes and therefore reduce the scope of the GREP query strategy. Increasing their number would probably lead to the collection of additional manuscripts to be read, which would be more time-consuming and therefore difficult to implement in routine diagnosis. An alternative could be to subscribe to RSS feeds from selected journals in the field of interest (Sobreira et al., 2015). This strategy requires the selection of appropriate journals and subsequent filtering of manuscripts of interest from these journals (Dubuque, 2011; Beller et al., 2018; Marshall and Wallace, 2019). Ultimately, the identification of manuscripts could be automated via a direct search in PubMed, which would facilitate the search and save time (Bohle, 2018). While retrieving manuscripts from appropriate journals is one component of the strategy, it must be acknowledged that systematic review of these manuscripts to identify the few manuscripts of interest is another. Indeed, identifying the name of a particular gene in a manuscript title does not imply that the article presents a novel gene–pathology association. To address this caveat, several text mining software tools have been developed, each with advantages and disadvantages (Rani et al., 2015; Van der Mierden et al., 2019).

In addition, updates to public and private databases could lead to update description of variants or changes in classification. Since a GREP query is performed on existing ES data, these variants, despite being detected, could be lacking crucial annotation for correct interpretation. The GREP query strategy only requires *a priori* knowledge of the genes of interest, since it is based on targeted querying of large-scale ES data. This GREP strategy also has great potential utility if collaborators send requests to one another regarding unpublished candidate genes in order to identify recurrences and establish phenotype–genotype correlations. For example, we identified strong candidate variants in one undiagnosed individual (namely, compound heterozygosity in *DOHH*, with molecular and clinical overlap) after a national collaborative call. However, whatever the mode of selection of the genes of interest, the effectiveness of the GREP strategy is all the greater when the disease is more heterogeneous, when the annual rate of identification of relevant new causal genes is high, and when large amounts of ES data are available. Accordingly, DD and ID are likely the best groups of rare diseases in which to apply this strategy. Nevertheless, efficient reanalysis strategies have also been reported in other rare disorders, such as sudden death, DD/ID, epilepsy, and Mendelian disorders (Costain et al., 2018; Ewans et al., 2018; Nambot et al., 2018; Baker et al., 2019; Li et al., 2019; Salfati et al., 2019).

Another advantage of the GREP query strategy is its ease of setup, since the GREP command-line tool is available in every Linux terminal. One disadvantage is the multiplicity of pipeline versions and/or reanalysis of ES data. Indeed, if different vcf files originating from the same affected individual, but with different versions of the pipeline (and most likely with additional annotations), are searched using a GREP query, then the output file resulting from the query will consist of multiples lines pertaining to the same variants (Figure 1B). Since vcf files can change in terms of the number of fields (due to the addition of novel *in silico* scores, databases, etc.), the duplicated lines for each variant will not present the same information; i.e., there will be missing information in some columns due to pipeline updates (Figure 1B). This issue is compounded when searches are carried out for multiple individuals who have benefited from different versions of the pipeline. Implementation of an in-house script can overcome this issue (Figure 1B), facilitating interpretation by decreasing variant redundancy in the results of the basic GREP query (with an 11-fold mean decrease between the basic and custom GREP query). However, even without a custom script, a basic GREP query can already provide files that are interpretable for biologists, and a GREP query remains easy to set up for routine use. The periodicity at which the GREP query was run (every day) was defined *a priori* in our study. While we could not define a mean time for the availability of manuscripts of interest in PubMed, the question of the periodicity of the PubMed inquiry is important, since a daily search will identify most of the relevant manuscripts but could be time-consuming. A monthly search would appear to be more practical in a diagnostic setting, with the drawback of the accumulation of manuscripts to either discard or retain. With this monthly periodicity, the OMIM update list could be used. However, this list is generally out of date: for example, the first new clinical synopses in January 2022 is based on a manuscript published in 2020 (a 2-year delay). In addition, the new entry list merely describes the relevant gene, without including the information that this gene is linked to a human disorder. Therefore, a clinician adopting this strategy would have to search every new entry to find out whether this is linked to a human disorder falling into their area of expertise.

Sometimes, several relevant manuscripts (up to four) are added to PubMed on the same day or on different days of the same week (up to seven manuscripts over 4 days of the same week), whereas sometimes no manuscripts are relevant for weeks (up to eight) at a time. Therefore, there is a fine line between setting a loose threshold that will miss some manuscripts and daily GREP querying that will lead to work overload. A compromise could be a one-off search capturing the most recent 20 articles or searches at two time points capturing 10 articles.

In conclusion, a reanalysis strategy combining intensive bibliographic monitoring and rapid GREP queries of a large ES database offers promising added value in increasing diagnostic yield and reducing diagnostic delay in rare diseases. Nevertheless, this strategy remains time-consuming, and automated bibliographic monitoring tools to pinpoint genes of interest will be welcomed, as these would lead to even faster diagnosis.

## Data Availability

The data presented in the study are deposited in the https://www.ncbi.nlm.nih.gov/clinvar/?term=SUB12859036 repository, accession number SUB12859036.
