# Supplementary material for: Combining globally search for a regular expression and print matching lines with bibliographic monitoring of genomic database improves diagnosis
Source: Front Genet. 2023 Apr 20;14:1122985. doi: 10.3389/fgene.2023.1122985 (PMC10157399; doi:10.3389/fgene.2023.1122985)
Supplement: Supplementary file 2 [file Table1.DOCX]

Supplementary Material

# Supplementary Figures and Tables

Table 1: Genes identified from manuscript of interest (with associated PMID) and by year.
